# Carotid intima-media thickness and flow-mediated dilation do not predict acute in-hospital outcomes in patients hospitalized with COVID-19

**DOI:** 10.1152/ajpheart.00026.2022

**Published:** 2022-03-25

**Authors:** Michelle Cristina-Oliveira, Kamila Meireles, Saulo Gil, Fábio Cavalcante Assis, João Carlos Geber-Júnior, Samuel Katsuyuki Shinjo, Heraldo Possolo de Souza, Alfredo Nicodemos Cruz Santana, Paul A. Swinton, Luciano F. Drager, Bruno Gualano, Hamilton Roschel, Tiago Peçanha

**Affiliations:** ^1^Applied Physiology and Nutrition Research Group, School of Physical Education and Sport, Faculdade de Medicina, Universidade de São Paulo, São Paulo, Brazil; ^2^Hospital das Clínicas, Faculdade de Medicina, Universidade de São Paulo, São Paulo, Brazil; ^3^Disciplina de Medicina de Emergência, Departamento de Clínica Médica, Faculdade de Medicina, Universidade de Brasília, Brasília, Brazil; ^4^Unidade de Terapia Intensiva Cardiológica, Departamento de Cardiopneumologia, Instituto do Coração, Hospital das Clinicas, Faculdade de Medicina, Universidade de São Paulo, São Paulo, Brazil; ^5^Escola Superior de Ciências da Saúde, Brasília, Brazil; ^6^School of Health Sciences, Robert Gordon University, Aberdeen, United Kingdom; ^7^Unidade de Hipertensão, Disciplina de Nefrologia e Instituto do Coracao, Hospital das Clinicas, Faculdade de Medicina, Universidade de São Paulo, São Paulo, Brazil; ^8^Institute of Sport, Department of Sport and Exercise Sciences, Faculty of Science and Engineering, Manchester Metropolitan University, Manchester, United Kingdom

**Keywords:** atherosclerosis, endothelium, mortality, SARS-CoV-2, thrombosis

## Abstract

Studies have suggested a potential role of endothelial dysfunction and atherosclerosis in the pathophysiology of COVID-19. Herein, we tested whether brachial flow-mediated dilation (FMD) and carotid intima-media thickness (cIMT) measured upon hospital admission are associated with acute in-hospital outcomes in patients hospitalized with COVID-19. A total of 211 patients hospitalized with COVID-19 were submitted to assessments of FMD and mean and maximum cIMT (cIMT_mean_ and cIMT_max_) within the first 72 h of hospital admission. Study primary outcome was a composite of intensive care unit admission, mechanical ventilation, or death during the hospitalization. These outcomes were also considered independently. Thrombotic events were included as a secondary outcome. Odds ratios (ORs) and confidence intervals (CIs) were calculated using unadjusted and adjusted multivariable logistic regression models. Eighty-eight (42%) participants demonstrated at least one of the composite outcomes. cIMT_mean_ and cIMT_max_ were predictors of mortality and thrombotic events in the univariate analysis (cIMT_mean_ and mortality: unadjusted OR 12.71 [95% CI 1.71–94.48]; *P* = 0.014; cIMT_mean_ and thrombotic events: unadjusted OR 11.94 [95% CI 1.64–86.79]; *P* = 0.015; cIMT_max_ and mortality: unadjusted OR 8.47 [95% CI 1.41–51.05]; *P* = 0.021; cIMT_max_ and thrombotic events: unadjusted OR 12.19 [95% CI 2.03–73.09]; *P* = 0.007). However, these associations were no longer present after adjustment for potential confounders (*P* > 0.05). In addition, FMD% was not associated with any outcome. In conclusion, cIMT and FMD are not independent predictors of clinical outcomes in patients hospitalized with COVID-19. These results suggest that subclinical atherosclerosis and endothelial dysfunction may not be the main drivers of COVID-19 complications in patients hospitalized with COVID-19.

**NEW & NOTEWORTHY** Studies have suggested a role of endothelial dysfunction and atherosclerosis in COVID-19 pathophysiology. In this prospective cohort study, we assessed the prognostic value of carotid intima-media thickness (IMT) and flow-mediated dilation (FMD) in patients with COVID-19. Carotid IMT and FMD were not independent predictors of major outcomes. These results suggest that other risk factors may be the main drivers of clinical outcomes in patients with COVID-19.

## INTRODUCTION

The novel coronavirus (COVID-19) is a viral disease caused by the severe acute respiratory syndrome coronavirus-2 (SARS-CoV-2). As of October 2021, COVID-19 has already infected over 240 million and killed around 5.0 million people across the globe, with Brazil being one the most severely hit country, totaling over 600,000 deaths and peaking at around 4,000 daily deaths (1). SARS-CoV-2 primarily affects the respiratory system, causing a wide range of symptoms, such as fever, dry cough, dyspnea, hypoxemia, and fatigue (2). Most patients with COVID-19 present with only mild upper respiratory tract infection; however, in the most severe cases, patients may require intensive care, with additional mechanical ventilatory support.

A hypercoagulable state is a common feature among patients with COVID-19 (3) and it is a critical contributor to the high morbidity and mortality in this disease (4). Incidence of venous and arterial thromboembolism in critical patients is estimated to be at around 20%–30% (4, 5). In addition, diffuse pulmonary microvascular thrombosis has been observed in autopsy studies of patients with COVID-19 (6), and maybe a contributor to progressive hypoxemia and requirements of mechanical ventilatory support in this disease (7). Part of the prothrombotic effect of the SARS-CoV-2 may involve a disruption in endothelium homeostasis. Indeed, evidence shows that the SARS-CoV-2 causes endothelial activation and dysfunction (6, 8), shifting the endothelial phenotype toward a prothrombotic, antifibrinolytic, and proinflammatory state (7). A case report including three patients with severe COVID-19 infection provided novel histological evidence of viral infection in endothelial cells and diffuse endothelial inflammation across multiple organ systems (8).

The hypothesis that endothelium damage may be pivotal to the complications associated with COVID-19 warrants further investigation. Brachial flow-mediated dilation (FMD) is a widely used method for the noninvasive assessment of endothelial function in conduit arteries in vivo (9). A reduced FMD is an early manifestation of vascular disease and atherosclerosis, and is an independent marker of atherothrombosis, and cardiovascular and all-cause mortality (10). FMD is also largely dependent on the production of nitric oxide (11), which is per se a potent antiplatelet and antithrombotic agent (12), and may predict complications in patients hospitalized with COVID-19-associated conditions, such as severe sepsis (13) and community-acquired pneumonia (14). Recent studies have also reported a reduction in FMD in patients with COVID-19 with different disease severities and at different times of the disease course (15–17). However, there is still limited information about the prognostic value of FMD for acute in-hospital outcomes in patients hospitalized with COVID-19.

In addition to FMD, assessment of carotid intima-media thickness (cIMT), a marker of subclinical atherosclerosis, has been used to predict future thrombotic events and overall mortality in multiple conditions (18). A recent study suggested that atherosclerosis may constitute an inflammatory environment predisposing to the aggravation of COVID-19 infection (19). Indeed, increased cIMT has been found in patients with systemic arterial hypertension, type 2 diabetes, and excess body weight (20), all of which have been linked with COVID-19 complications. A recent case series of three patients with COVID-19 with fulminant carotid thrombosis overlying previously mild carotid plaques and intima thickening, open the perspective that cIMT may also provide prognostic information on COVID-19-related complications and mortality (21), which needs to be further explored.

As the endothelial dysfunction and atherosclerosis may contribute to the severity of COVID-19 infection, the present study aimed to assess the associations between FMD and cIMT with COVID-19-related complications and mortality in patients hospitalized with COVID-19. The study hypothesis was that FMD and cIMT would be associated with clinical outcomes in patients hospitalized with COVID-19.

## METHODS

### Study Population and Design

This is a prospective cohort study conducted at the Clinical Hospital of the School of Medicine of the University of São Paulo (HCFMUSP), Sao Paulo, Brazil. This study protocol was registered at ClinicalTrials.gov (NCT04714125) and the manuscript was reported according to the Strengthening the Reporting of Observational Studies in Epidemiology (STROBE) checklist (Supplementary Fig. S1; https://doi.org/10.6084/m9.figshare.19323335) (22). Data reported herein were collected between June 2020 and May 2021.

Patients recently admitted to the hospital were recruited at the emergency department and outpatient clinics at the HCFMUSP. Inclusion criteria were ≥18 yr; admitted to hospital in ≤72 h and not yet admitted to the intensive care unit (ICU); and diagnosis of COVID-19 on the basis of a positive SARS-CoV-2 PCR test or a clinical diagnosis of COVID-19 with a negative PCR (or no swab taken) if they had typical clinical, radiological [bilateral multifocal ground-glass opacities (CT)], serological (IgG antibodies against SARS-CoV-2), and biochemical features consistent with COVID-19 and had been treated as COVID-19. Participants in a delirium state or with a recent history of endotracheal intubation were not included in the present study. Before participation, eligible participants received a detailed explanation of the experimental procedures and provided their written informed consent. The study followed the principles of the Declaration of Helsinki and was approved by the local Institutional Ethics Committee (Reference No. 4.243.688/2020).

### Data Collection

Participants were evaluated at the point of care within the first 72 h of hospital admission. Demographic and clinical data were collected through personal interviews and medical records. Subsequently, patients underwent cIMT and brachial FMD evaluations, which were undertaken by an experienced evaluator blinded to the participant’s clinical history. Participants were followed until hospital discharge or death, and outcomes were constantly monitored through medical records.

FMD was evaluated according to current guidelines (9) using a high-resolution ultrasound machine (LOGIQ e PRO, GE Healthcare, Chicago, IL) equipped with a 4.0–12.0-MHz linear transducer. The examination was performed in the opposite arm/forearm of the side where vascular access was located. Initially, participants were positioned in the supine position with their arm extended at an angle of ∼80° from the torso. A pneumatic cuff was positioned at the participants’ forearm to provide the ischemic stimulus. Longitudinal images of the brachial artery diameter were taken using the B-mode ultrasound, and simultaneous pulse-waved Doppler blood flow velocity was obtained using a 60° insonation angle with the sample volume placed in midartery and aligned with the blood flow. Initially, a 1-min baseline recording of the brachial artery diameter and blood flow velocity was performed and then the forearm cuff was inflated (∼200 mmHg) for 5 min. Recordings were resumed 30 s before cuff deflation and continued for 3 min thereafter. Brachial artery diameter and shear rate (4 × mean blood velocity/internal diameter) were analyzed by a blinded evaluator using a semiautomatic edge-detection and wall-tracking software (Cardiovascular Suite, Quipu, Italy). FMD% was calculated as the percentage change of the vessel diameter after cuff release in relation to baseline vessel diameter [FMD = (*D*_max_ – *D*_baseline_/*D*_baseline_) × 100]. To describe the relevant shear rate stimulus for FMD, we also calculated the area under the curve of the shear rate up to the peak diameter (SRAUC). Other parameters obtained from the FMD evaluation, including brachial artery baseline diameter, peak diameter, absolute dilation (FMD_abs_), and time-to-peak diameter were also calculated and reported (9). Finally, the function of downstream resistance vessels was assessed through the calculation of the peak blood velocity during reactive hyperemia (peak blood velocity) and the area under the curve of the blood velocity across the 3 min of postocclusive reactive hyperemia (VRH) (23).

cIMT was evaluated according to current guidelines (24) using a high-resolution ultrasound machine (LOGIQ e PRO, GE Healthcare, Chicago, IL), with the participants in supine position and semiextended neck. A linear transducer (12 L-RS, 4.0–12.0 MHz) was positioned at the participants’ right or left neck (the same side as the FMD) and the respective common carotid artery was insonated longitudinally 1 to 2 cm below the bifurcation, using the lateral imaging plane. Images were recorded for 15–20 s using the B-mode of the ultrasound. Thereafter, cIMT was analyzed by a blinded evaluator using a semiautomatic edge-detection and wall-tracking software (Cardiovascular Suite, Quipu, Italy). cIMT was measured at the distal wall of the carotid artery on a ∼10-mm segment, from the lumen-intima interface to the media-adventitia interface. The mean (cIMT_mean_, i.e., the average thickness of the entire segment) and maximal cIMT (cIMT_max_, i.e., the point of maximum thickness of the segment) across the 10-mm segment were measured in three “frozen” end-diastolic vessel images taken from the 15-s video and calculated as the average of the three images.

### Outcomes

The primary outcome was a composite of ICU admission, mechanical ventilation, or death during the period of hospitalization. These outcomes were also considered independently. In addition, thrombotic events, which comprised pulmonary embolism, deep-vein thrombosis, ischemic stroke, myocardial infarction, or systemic arterial embolism, were included as a secondary outcome.

### Statistical Analysis

Analyses were performed in the statistical environment R (v. 3.6.1, R Core Team 2021). An a priori estimation of sample size requirements and total number of predictors for logistic regression was conducted to achieve small optimism in predictor effect estimates as defined by a global shrinkage factor of 0.9 (25). Based on an *R*^2^ value of 0.30 (26) and a selection of eight predictors, it was estimated that a sample size of 197 participants was required (25). An independent Student’s *t* test or a Mann–Whitney *U* test was used, whenever appropriate, to compare clinical data, FMD, and cIMT parameters between participants presenting or not the composite outcome. A χ^2^ test was used to compare the categorical variables between groups. The association of FMD% and cIMT with primary (ICU admission, intubation, or mortality) and secondary (thrombotic events) end points were quantified by unadjusted and adjusted multivariable logistic regression. Two sets of adjusted models were investigated with the first controlling for sex, age, blood oxygen saturation at hospital’s admission ($\text{S}{\text{a}}_{{\text{O}}_{2}}$%), smoking, obesity, and preexisting condition (binary variable including cardiometabolic and pulmonary conditions, e.g., type 2 diabetes, systemic arterial hypertension, cardiovascular diseases, previous cardiac surgery, chronic kidney disease, chronic obstructive pulmonary disease); the second included the same covariates as the first and also included serum D-dimer and C-reactive protein (CRP) levels. Missing data (proportion missing: FMD% = 18%; serum D-dimer = 16.7%; cIMT = 13%; CRP = 10%) were imputed using multiple imputation (*m* = 10) with all available data and the “mice” package (27) with variance in pooled regression analyses accounting for uncertainty in missing value imputation. Model fit was quantified with Nagelkerke *R*^2^ with division by the maximum attainable *R*^2^ value. For all tests, the significance level was set at 5%. Continuous data are presented as means ± SD or median ± interquartile range (IQR), and categorical data are presented as percentages.

## RESULTS

Between June 2020 and May 2021, 631 patients admitted to the emergency department with suspected COVID-19 were screened for participation, and 262 presented the study criteria and were accepted to participate in the study. However, 51 of them did not have a confirmed COVID-19 diagnosis and, therefore, were excluded from the study. Therefore, 211 participants were included in the final analysis. The clinical characteristics of the study participants are presented in Table 1. Overall, the most prevalent comorbidities were systemic arterial hypertension (59%), diabetes mellitus (37%), obesity (30%), and chronic kidney disease (15%).

**Table 1.** Clinical characteristics of the study participants

		Composite Outcome (ICU, Mechanical Ventilation, or Death)		
	All Participants	No	Yes	*P*-Value
*n*	211	123	88	
Age, yr	58 ± 16	57 ± 16	60 ± 16	0.104
Female, *n* (%)	104 (49)	65 (53)	39 (44)	0.279
Smoking, *n* (%)	21 (10)	12 (10)	9 (10)	1.000
BMI, kg/m^2^	30.0 ± 8.6	29.4 ± 8.3	30.5 ± 8.8	0.474
Comorbidities on admission, *n* (%)				
Obesity	63 (30)	35 (28)	28 (32)	0.796
Hypertension	124 (59)	68 (55)	56 (63)	0.283
DM	78 (37)	40 (33)	38 (43)	0.150
Asthma	12 (6)	7 (6)	5 (6)	1.000
COPD	12 (6)	7 (6)	5 (6)	1.000
CAD	19 (9)	9 (7)	10 (11)	0.442
CKD	32 (15)	13 (11)	19 (22)	0.044
AMI	13 (6)	7 (6)	6 (7)	0.963
Malignancies	21 (10)	13 (11)	8 (9)	0.916
Autoimmune diseases	12 (6)	8 (7)	4 (5)	0.769
Liver transplant	3 (1)	3 (2)	2 (2)	0.378
Kidney transplant	14 (7)	5 (4)	9 (10)	0.131
Lung transplant	1 (<1)	1 (1)	2 (2)	1.00
Presentation on admission				
Body temperature, °C	36.2 ± 2.4	36.2 ± 3.1	36.3 ± 0.9	0.612
$\text{S}{\text{a}}_{{\text{O}}_{2}}$%	94 ± 4	94 ± 4	94 ± 4	0.394
Systolic BP, mmHg	127 ± 17	127 ± 17	126 ± 18	0.623
Diastolic BP, mmHg	77 ± 12	77 ± 12	77 ± 12	0.757
Initial laboratory markers				
Creatinine, mg/dL	0.9 [0.7–1.3]	0.9 [0.7–1.2]	1.0 [0.8–1.5]	0.224
CRP, mg/L	78 [39–139]	72 [30–112]	87 [55–179]***	0.001
Hemoglobin, g/L	12.8 [11.2–14.1]	12.8 [11.4–14.1]	12.9 [11.1–14.2]	0.302
Platelet, ×10^3^/mm^3^	218 [162–300]	219 [159–300]	217 [168–299]	0.479
D-dimer, ng/mL	975 [608–2,114]	949 [535–2,086]	1,076 [736–2,171]	0.739
Troponin, ng/mL	0.012 [0.007–0.027]	0.009 [0.006–0.020]	0.016 [0.008–0.047]	0.375

Values are means ± SD or [interquartile range]. AMI, acute myocardial infarction; BMI, body mass index; BP, blood pressure. CAD, coronary artery disease. CKD, chronic kidney disease. COPD, chronic obstructive pulmonary disease; CRP, C-reactive protein; DM, diabetes mellitus; $\text{S}{\text{a}}_{{\text{O}}_{2}}$%, blood oxygen saturation at hospital’s admission. Continuous data are presented as means ± SD (age, BMI, body temperature, and $\text{S}{\text{a}}_{{\text{O}}_{2}}$%) or median (interquartile range) (initial laboratory markers). Categorical data are presented as counts and percentages. ***P ≤ 0.001 between groups.

Mean hospital length of stay was 12 (±13) days. During this period, 80 (38%) participants were admitted to ICU, 40 (19%) required mechanical ventilatory support, and 35 (17%) died. In total, 88 patients (42%) presented at least one of the composite outcomes. In addition, 35 (17%) participants had a thrombotic event during the period of hospitalization.

Participants with at least one of the composite outcomes had higher serum levels of CRP compared with the participants who did not present the composite outcomes. However, there was no significant difference between groups in any other clinical outcome (Table 1).

**Figure 1.**
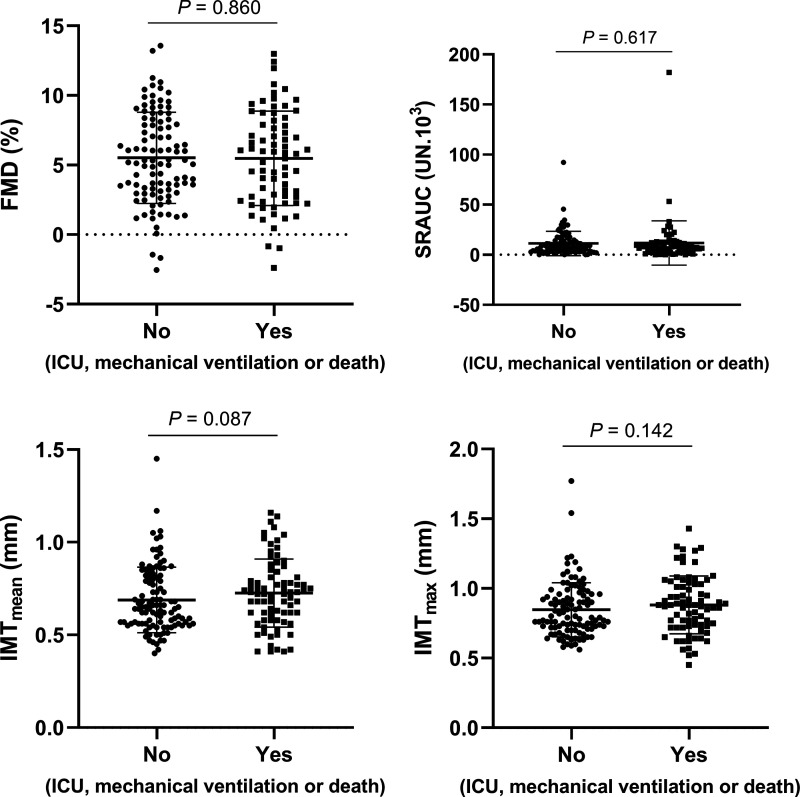
Brachial artery flow-mediated dilation (FMD%), area under the curve of the shear rate up to the peak diameter (SRAUC), mean common carotid intima-media thickness (cIMT_mean_), and maximum common carotid intima-media thickness (cIMT_max_) in patients with COVID-19 that presented or not a composite of intensive care unit (ICU) admission, mechanical ventilation, or death during the period of hospitalization. *n* values: FMD%, No = 101/Yes = 72; SRAUC, No = 97/Yes = 72; cIMT_mean_, No = 107/Yes = 77; cIMT_max_, No = 107/Yes = 77. Data were compared by independent Student’s *t* test with imputed data.

Among all participants, we were able to obtain high-quality recordings of FMD and cIMT in 173 and 184 patients, respectively. There were no differences in FMD (5.45 ± 3.42 vs. 5.66 ± 3.38%, *P* = 0.860), SRAUC (13.60 ± 27.27 vs. 10.70 ± 11.50 UN·10^3^; *P* = 0.617), cIMT_mean_ (0.73 ± 0.19 vs. 0.68 ± 0.17 mm, *P* = 0.087), and cIMT_max_ (0.89 ± 0.21 vs. 0.84 ± 0.19 mm, *P* = 0.142) between patients with COVID-19 presenting or not the composite outcome (Fig. 1). The additional parameters of the FMD test were also not different between patients with COVID-19 presenting or not the composite outcome (Table 2).

**Table 2.** Additional parameters of flow-mediated dilation test

		Composite Outcome (ICU, Mechanical Ventilation, or Death)		
	All Participants	No	Yes	*P* Value
*n*	211	123	88	
Baseline diameter, mm	4.43 ± 0.80	4.36 ± 0.79	4.53 ± 0.79	0.076
Peak diameter, mm	4.67 ± 0.80	4.60 ± 0.80	4.77 ± 0.80	0.069
FMD_abs_, mm	0.24 ± 0.14	0.24 ± 0.14	0.24 ± 0.14	0.735
Time to peak, s	84.59 ± 43.26	79.33 ± 41.4	91.94 ± 44.95	0.093
Peak blood velocity, cm/s^2^	3.84 ± 0.56	3.84 ± 0.58	3.85 ± 0.54	0.906
VRH, cm	7.92 ± 0.70	7.83 ± 0.72	8.04 ± 0.64	0.124

Values are means ± SD. FMD_abs_, absolute flow-mediated dilation; peak blood velocity, peak blood velocity during postocclusive reactive hyperemia; time to peak, time to peak dilation; VRH, area under the curve of the blood velocity across the 3 min of postocclusive reactive hyperemia.

Table 3 summarizes the results of logistic regression analysis (odds ratio and associated 95% confidence intervals) unadjusted and adjusted for sex, age, $\text{S}{\text{a}}_{{\text{O}}_{2}}$%, smoking, obesity, and preexisting conditions (*model 1*), and by these same previous covariates with the addition of D-dimer and CRP levels (*model 2*). In the unadjusted analysis, cIMT_mean_ was a significant predictor of mortality (unadjusted OR, 12.71 [95% CI, 1.71–94.48]; *P* = 0.014) and thrombotic events (unadjusted OR, 11.94 [95% CI, 1.64–86.79]; *P* = 0.015). However, addition of covariates increased the uncertainty of these associations (cIMT_mean_ and mortality: *P* = 0.590–0.747; cIMT_mean_ and thrombotic events, *P* = 0.121–0.172). Similarly, in the unadjusted analysis, cIMT_max_ was a significant predictor of mortality (unadjusted OR, 8.47 [95% CI, 1.41–51.05]; *P* = 0.021) and thrombotic events (unadjusted OR, 12.19 [95% CI, 2.03–73.09]; *P* = 0.007). However, addition of covariates also reduced the significance of these associations (cIMT_max_ and mortality: *P* = 0.621–0.741; cIMT_max_ and thrombotic events, *P* = 0.051–0.076). FMD% was not a significant predictor of any clinical end point (*P* = 0.067–0.710 in the multivariable analyses).

**Table 3.** Associations [odds ratio (95% CI)] of FMD%, cIMT_mean_, and cIMT_max_ with clinical end points

	Unadjusted Coefficient			Adjusted *Model 1* Coefficient‡			Adjusted *Model 2* Coefficient¥		
	OR [95% CI]	*P* Value	Model Summary *R*^2^	OR [95% CI]	*P* Value	Model Summary *R*^2^	OR [95% CI]	*P V*alue	Model Summary *R*^2^
Composite outcome†									
FMD, %	0.99 [0.91–1.09]	0.909	<0.01	1.03 [0.93–1.13]	0.594	0.07	1.03 [0.92–1.15]	0.633	0.14
cIMT_mean,_ mm	3.76 [0.74–19.22]	0.113	0.02	1.20 [0.13–11.03]	0.874	0.06	1.78 [0.18–18.11]	0.626	0.12
cIMT_max,_ mm	2.78 [0.63–12.19]	0.177	0.01	1.01 [0.15–6.98]	0.989	0.06	1.46 [0.19–11.27]	0.719	0.12
Mortality									
FMD, %	1.01 [0.90–1.14]	0.858	<0.01	1.10 [0.95–1.27]	0.211	0.22	1.08 [0.93–1.26]	0.303	0.24
cIMT_mean,_ mm	12.71 [1.71–94.48]	0.014	0.05	1.57 [0.10–24.57]	0.747	0.20	2.20 [0.12–38.86]	0.590	0.24
cIMT_max,_ mm	8.47 [1.41–51.05]	0.021	0.04	1.50 [0.13–16.74]	0.741	0.20	1.89 [0.15–23.77]	0.621	0.24
ICU admission									
FMD, %	0.96 [0.87–1.05]	0.347	<0.00	0.98 [0.89–1.08]	0.661	0.05	0.97 [0.87–1.09]	0.634	0.14
cIMT_mean,_ mm	1.29 [0.26–6.41]	0.758	<0.00	0.56 [0.06–5.16]	0.610	0.05	0.74 [0.07–8.05]	0.804	0.11
cIMT_max,_ mm	1.01 [0.23–4.41]	0.985	<0.00	0.47 [0.06–3.40]	0.454	0.05	0.60 [0.07–5.15]	0.644	0.11
Mechanical ventilation									
FMD, %	1.00 [0.90–1.12]	0.980	<0.01	1.04 [0.91–1.19]	0.592	0.13	1.03 [0.90–1.18]	0.710	0.16
cIMT_mean,_ mm	1.84 [0.26–12.89]	0.538	0.01	1.08 [0.08–15.19]	0.956	0.13	1.49 [0.09–24.02]	0.779	0.17
cIMT_max,_ mm	1.26 [0.21–7.54]	0.800	<0.01	0.73 [0.07–7.63]	0.792	0.13	0.90 [0.08–10.58]	0.934	0.18
Thrombotic events									
FMD, %	1.09 [0.95–1.25]	0.252	<0.01	1.14 [0.98–1.33]	0.069	0.11	1.15 [0.99–1.34]	0.067	0.14
cIMT_mean,_ mm	11.94 [1.64–86.79]	0.015	0.05	8.01 [0.58–110.21]	0.121	0.11	6.75 [0.44–103.52]	0.172	0.12
cIMT_max,_ mm	12.19 [2.03–73.09]	0.007	0.06	9.77 [0.99–95.01]	0.051	0.12	8.55 [0.81–90.28]	0.076	0.13

Values are odds ratio (OR) [95% confidence interval (CI)]. cIMT_mean_, mean-mean common carotid intima-media thickness; cIMT_max_; mean-maximum common carotid intima-media thickness; FMD%, brachial artery flow-mediated dilation. †Composite outcome included requirements of ICU, mechanical ventilation, and/or death. ‡Adjusted *model 1* was controlled for sex, age, blood oxygen saturation ($\text{S}{\text{a}}_{{\text{O}}_{2}}$%) at hospital’s admission, smoking, obesity, and preexisting condition (binary variable including cardiometabolic and pulmonary conditions, e.g., diabetes, hypertension, cardiovascular diseases, previous cardiac surgery, chronic kidney disease, chronic obstructive pulmonary disease). ¥Adjusted *model 2* was controlled for all variables of *model 1* and also included serum D-dimer and CRP levels.

## DISCUSSION

This prospective cohort study tested the associations between FMD and cIMT with clinical outcomes in patients hospitalized with COVID-19. We found that cIMT_mean_ and cIMT_max_ were both predictors of mortality and thrombotic events in patients with COVID-19 in the univariate analysis. However, adjustment for potential confounding factors reduced the magnitude of these associations and the certainty of the results. In addition, contrary to our hypothesis, FMD was not associated with any clinical end point.

Increased cIMT is an indicator of subclinical atherosclerosis and is independently associated with higher rates of atherothrombotic events (18). In addition, previous studies have suggested that atherosclerosis, a chronic inflammatory disease, may offer an ideal environment for the worsening of COVID-19 infection (19). Indeed, in atherosclerosis, diverse proinflammatory pathways are hyperactivated (e.g., TLR4/NF-κβ) and cytokines are chronically overexpressed (28), which may amplify the immune-mediated response to the SARS-CoV-2, increasing the susceptibility to a cytokine storm, and plaque rupture and thrombosis. Based on this rationale, one of the hypotheses of the present study was that increased cIMT would also be associated with COVID-19-related outcomes. In the present study, cIMT_mean_ and cIMT_max_ were predictors of mortality and thrombotic events in the univariate analysis; however, these associations were weak and no longer present after adjustment for potential confounders, which suggests that associations between cIMT and COVID-19 outcomes may be mediated by co-occurring risk factors such as obesity, existing cardiometabolic and pulmonary conditions, and CRP and D-dimer.

Contrary to our initial hypothesis, FMD was not a predictor of any major outcome. As post hoc analysis, we have also tested the associations between resistance vessel function parameters (peak blood velocity and VRH) and the study outcomes, and we found no consistent associations between these variables (Supplementary Table S1; https://doi.org/10.6084/m9.figshare.19375292). These findings diverge from studies reporting a central role of endothelial dysfunction in the COVID-19-related complications (6–8). For instance, patients with severe sepsis showed reduced FMD in comparison with healthy controls, and reduced brachial hyperemic response to ischemia, which was associated with hospital mortality (13). In addition, in patients with community-acquired pneumonia, a reduced FMD upon hospitalization was inversely associated with disease severity and serum endotoxins (14). A recent study has also shown that patients hospitalized with COVID-19 present reduced FMD compared with patients hospitalized without COVID-19; patients with COVID-19 and with FMD lower than or equal to 3.43% remained in the hospital longer, required more oxygen supplementation, and had a higher mortality rate than patients with higher FMD (16). Ratchford et al. (17) also reported reduced FMD in young adults with mild cases of COVID-19 compared with healthy subjects. These reductions in FMD have also been reported in persistently symptomatic adults who were beyond the acute phase of the COVID-19 disease (15). Surprisingly, in the present study, the mean FMD was 5.7%, which is higher than those observed in the aforementioned studies (FMD <3%–4%) and also falls in the “normality range” for FMD in healthy middle-aged and elderly individuals (5.11%–6.12%) (29). However, a reanalysis of the present study data using FMD < 3.43% as a threshold for endothelial dysfunction also did not observe associations between FMD and any of the study outcomes (data not shown). Differences in the time of the FMD measurement may partially explain the discrepant results. In the present study, patients were assessed <72 h after hospitalization, which coincides with the beginning of more severe symptoms for most of them; conversely, in the two previous studies, FMD assessments were taken 5–25 days after symptoms onset. Taken together, these findings suggest that FMD deterioration may continuously progress throughout the course of the infection and that most of our participants may have been in earlier stages by the time FMD was assessed. Future studies should investigate the potential FMD decay during infection, and its prognostic value for poor outcomes among patients with COVID-19.

The findings of the present study do not contest the importance of vascular health for COVID-19 prognosis. Instead, it suggests that alterations in the vascular phenotype are possibly not the main drive, but a consequence of the presence of other risk factors that may per se affect COVID-19 progression in patients who were hospitalized. For instance, age, smoking, obesity, and preexisting diseases have been strongly associated with both COVID-19 events (30) and increased cIMT (20, 31), which may help to explain the significant associations between cIMT and mortality in the unadjusted, but not in the adjusted analyses. The data from the present study suggest that a proper control of modifiable risk factors (e.g., obesity, hypertension, diabetes) may offer the best protection against poor COVID-19 outcomes, and may slow the atherosclerosis progression, which could also be desirable beyond the COVID-19 infection.

The present study is the largest to investigate the prognostic value of ultrasound vascular measures in patients hospitalized with COVID-19. It is also worth mentioning that this study was conducted in Brazil, which is one of the world’s worst-affected countries by the pandemic, with a continuing high number of active positive cases and hospitalizations due to COVID-19. This study also presents some limitations. First, the observational nature of the study does not allow causality to be inferred. It is also not possible to accurately determine the exact time since infection and assessment in our participants, as they were admitted to our referring hospital days after the first diagnosis/symptoms (e.g., usually when persistent moderate-to-severe symptoms were present). In addition, patients were receiving different medications at the time of hospitalization and received different treatment regimens for COVID-19 during the hospitalization period, and some of these medications might have affected associations between the study predictors and outcomes. Moreover, although we have followed current guidelines for the assessment of both cIMT and FMD, some recommendations for participants’ preparation, such as standardizing the time of the day of measurement, and the time after food and drug intake, were not possible due to competing priorities within the healthcare setting. Nonadherence to such aspects may have decreased FMD reproducibility (9), which may also help to explain the absence of association between FMD and study outcomes. Importantly, although our study is powered to detect changes in the selected outcomes, this was still a small and heterogeneous cohort, which may help to explain the wide confidence intervals and preclude potential subgroup comparisons and further adjustments for other covariates. Finally, data reported herein are limited to acute in-hospital outcomes, and to patients hospitalized with COVID-19 that accepted to have their physiological data collected while having a symptomatic infection and during an unprecedented pandemic. It is likely that different results could have been obtained in a more diverse COVID-19 population and with long-term outcomes.

In conclusion, mean and maximal cIMT assessed upon hospital admission are not independently associated with acute in-hospital outcomes in patients hospitalized with COVID-19. Increased cIMT_mean_ and cIMT_max_ were associated with higher odds of mortality and thrombotic events in the univariate analysis only, which suggest that other risk factors must be more important for the prognosis of patients hospitalized with COVID-19. In addition, FMD, a marker of endothelial dysfunction, was not consistently associated with any study outcome. Taken together, these results suggest that subclinical atherosclerosis may not be the main driver of COVID-19 complications in hospitalized patients with COVID-19. In addition, the results of the present study do not support the assessment of atherosclerotic risk to identify patients with COVID-19 prone to poor prognosis.

## SUPPLEMENTAL DATA

10.6084/m9.figshare.19323335 Supplemental Figure S1: https://doi.org/10.6084/m9.figshare.19323335

10.6084/m9.figshare.19375292 Table S1: https://doi.org/10.6084/m9.figshare.19375292.

## GRANTS

This work was supported by Fundação de Amparo à Pesquisa do Estado de São Paulo Grants 2016/23319-0 and 2017/13552-2 and Conselho Nacional de Desenvolvimento Científico e Tecnológico Grants 406196/2018-4 and 428242/2018-9.

## DISCLOSURES

No conflicts of interest, financial or otherwise, are declared by the authors.

## AUTHOR CONTRIBUTIONS

M.C.-O., F.C.d.A., S.K.S., H.P.d.S., A.N.d.C.S., L.F.D., B.G., H.R., and T.P. conceived and designed research; M.C.-O., K.M., and F.C.d.A. performed experiments; M.C.-O., K.M., S.G., J.C.G.-J., and P.A.S. analyzed data; K.M., S.G., J.C.G.-J., S.K.S., P.A.S., L.F.D., B.G., H.R., and T.P. interpreted results of experiments; P.A.S. and T.P. prepared figures; S.K.S., P.A.S., L.F.D., B.G., H.R., and T.P. drafted manuscript; M.C.-O., K.M., S.G., F.C.d.A., J.C.G.-J., S.K.S., H.P.d.S., A.N.d.C.S., P.A.S., L.F.D., B.G., H.R., and T.P. edited and revised manuscript; M.C.-O., K.M., S.G., F.C.d.A., J.C.G.-J., S.K.S., H.P.d.S., A.N.d.C.S., P.A.S., L.F.D., B.G., H.R., and T.P. approved final version of manuscript.
